# Synthesis and evaluation of Cd-Co_3_O_4_@SGCN nanocomposites for photocatalytic degradation of RY-18 and inactivation of pathogenic bacteria

**DOI:** 10.3389/fchem.2026.1896769

**Published:** 2026-08-24

**Authors:** Ammara, Abdur Rauf, Sana Iqbal, Lubna Rasool, Vallel Zahra, Mohsin Javed, Faizan Iqbal, Adeeb Shehzad, Ali Bahadur, Shahid Iqbal, Sajid Mahmood, Shaimaa A. M. Abdelmohsen, Renad Al-Ghamdi

**Affiliations:** 1 Department of Chemistry, University of Sahiwal, Sahiwal, Pakistan; 2 Department of Chemistry, School of Science, University of Management and Technology, Lahore, Pakistan; 3 Research Center, Dhofar University, Salalah, Oman; 4 Department of Chemistry, Nanomaterials Research Center, College of Science, Mathematics and Technology, Wenzhou-Kean University, Wenzhou, Zhejiang, China; 5 Dorothy and George Hennings College of Science, Mathematics and Technology, Kean University, Union, NJ, United States; 6 Department of Chemical and Environmental Engineering, University of Nottingham Ningbo China, Ningbo, China; 7 Low Dimensional Materials Research Center at Khazar University, Baku, Azerbaijan; 8 Department of Physics, College of Science, Princess Nourah bint Abdulrahman University, Riyadh, Saudi Arabia; 9 Graduate of Department of Epidemiology, College of Health and Rehabilitation Sciences, Princess Nourah bint Abdulrahman University, Riyadh, Saudi Arabia

**Keywords:** adsorption, antibacterial activity, photocatalysis, Reactive Yellow 18 (RY-18), SGCN (sulphur doped graphitic carbon nitride) Cd-Co3O4@SGCN

## Abstract

In this research, cobalt oxide (Co_3_O_4_) nanoparticles and a series (1.3–9.3 mol%) of cadmium doped cobalt oxide (Cd-Co_3_O_4_) were synthesized using a simple co-precipitation method. All the prepared nanoparticles were characterized by FTIR, XRD, SEM, EDX and UV-Visible spectroscopy. The crystalline structure of Co_3_O_4_ was verified by XRD analysis, and the material’s structural and electrical characteristics were successfully altered by Cd inclusion. The degradation efficiency of RY-18 decreased gradually with the increase of Cd concentration, and reached the maximum efficiency of 68% for 5.3% Cd-Co_3_O_4_, after 150 min of sunlight irradiation, compared to 33% for pure Co_3_O_4_. The photocatalytic efficiency was then reduced when the concentration of Cd was raised further (7.3% and 9.3%) because of the excessive electron–hole recombination induced by the existence of Cd. To further improve the photocatalytic activity, 5.3% Cd-Co_3_O_4_ was coupled with optimized 10%–70% SGCN and the 5.3% Cd-Co_3_O_4_@50% SGCN nanocomposite showed highest degradation performance because of improved light absorption and efficient charge transfer between the two components. Additionally, synthesized nanoparticles and nanocomposites exhibited antibacterial properties, which could be applied as antibacterial multifunctional materials in environmental remediation applications.

## 1 Introduction

Water is a crucial resource for human civilization and the most essential substance for life on Earth. Ensuring a steady supply of affordable and clean water is a key humanitarian goal. Water contamination is a major global concern due to rapid urbanization and industrialization (Kathing and Saini, 2022; Khan et al., 2019; Rathod et al., 2024). It is mainly caused by organic pollutants like industrial dyes and agricultural waste, which harm ecosystems and human health (Bouras et al., 2023; Bukhamsin et al., 2024; Dada et al., 2024). According to estimates from the World Health Organization (WHO), diseases including cholera, hepatitis, typhoid, diarrhea, and cancer caused by water pollution claim the lives of 2.3 million people annually (Riaz et al., 2023; Shaban et al., 2018). Dyes are the leading source of environmental pollution among water contaminants because of their toxicity and lack of biodegradability (Mousa et al., 2024). Annually over 70,000 tons of about 10,000 different colors and shades are distributed worldwide (Al et al., 2022; Ali et al., 2023; Hassan Ibrahim et al., 2024; Kishor et al., 2021). Additionally; roughly 280,000 tons of toxic dyes are discharged into water bodies each year by the textile, paper, plastic and rubber industries (Shahzadi et al., 2022).

Due to their structure and intended use, dyes are classified as acidic, basic, reactive, vat and azoic dyes (Al et al., 2022; Moridon et al., 2019; Supin et al., 2024). Reactive azo dyes are most used because of their high stability during washing and low cost. Additionally, because of their high durability and intricate aromatic structures, which make removal challenging, more than 15% of reactive azo dyes wind up in water and cause major environmental issues (Hassanin et al., 2021; Xiao et al., 2023). Reactive Yellow 18 (RY-18), an anionic reactive azo dye, is extensively used in the textile industry. Therefore, many efforts have been made by researchers to eliminate these dyes from the environment before they are released, using various techniques such as physical, chemical and biological degradation (Wahab et al., 2021). Photocatalytic degradation has attracted significant interest because of its benefits, including non-selective oxidation, affordability and ease of control. It is also recognized as a sustainable, energy-efficient and environmentally friendly technology. Numerous semiconductor photocatalysts, such as SnO_2_ (Abdullah et al., 2018; Kumarage and Comini, 2021; Srivastava et al., 2022), CdO (Lakra et al., 2021), ZnO (Mahmood et al., 2022), Co_3_O_4_ (Liang et al., 2025), TiO2 (Wei et al., 2022) and CuO (Abdulwahab et al., 2023) have been studied. However, their efficacy in visible-light photocatalysis is limited by their large band gaps and quick electron-hole recombination.

Cobalt oxide (Co_3_O_4_), a p-type semiconductor has a wide band gap gained more attention recently for the photodegradation of dye pollutants because of its high surface-to-volume ratio, affordability, environmental friendliness, ease of preparation and superior chemical and physical stability (Hassanin et al., 2021; Moridon et al., 2019; Supin et al., 2024). Due to the exceptional optical, electrical and magnetic properties of Co_3_O_4_ nanomaterial, it has also been used in a variety of devices, including energy storage (Xiao et al., 2023), gas sensors (Abdullah et al., 2018; Kumarage and Comini, 2021; Wahab et al., 2021), supercapacitors (Lakra et al., 2021; Srivastava et al., 2022), lithium-ion batteries (Mahmood et al., 2022) and CO oxidation at low temperatures (Liang et al., 2025). Moreover, Co_3_O_4_ has several disadvantages including photo corrosion, visible light inactivity and the quick recombination of photogenerated electron-hole pairs. Due to quick e^−^ and h^+^ pair recombination few electrons are moved to the pollutants conduction band. The practical application of Co_3_O_4_ in photocatalytic processes is hindered by these issues.

Various techniques have been emerging to narrow the bandgap, such as doping of non-metal or metal and preparing composites by using a suitable dopant (Abdulwahab et al., 2023; Medhi et al., 2020; Suresh et al., 2023; Wang et al., 2022; Wei et al., 2022). In the bandgap of a photocatalyst, these dopants supply impurities and generate local energy levels that yield free charge carriers, which initiate the surface chemical reaction and produce an invisible light response. Faisal et al. reported zinc-doped cobalt oxide as a proficient photocatalyst for the removal of malachite green (96%) after 100 min of irradiation with sunlight (Faisal et al., 2022). Tamil et al. studied the chitosan-based nanocomposites of Cu doped cobalt oxide as an effective antibacterial agent and photocatalyst for dye removal (Thendral et al., 2024). Hitkari et al. investigated Cr doped cobalt oxide by coprecipitation for the degradation of methylene blue (Hitkari et al., 2017). Co_3_O_4_/Sn-ZnO nanocomposite reported by Zena et al. for elimination of methylene blue dye (95.1%) by the Sol-gel method (Zena et al., 2024). Cadmium and Graphitic carbon nitride are potential candidates as a dopant. The cubic structure of cadmium oxide (CdO) and its narrow (2.3 eV) direct band gap allow for the modification of Co_3_O_4_ structural, optical, electrical and chemical characteristics through Cd-doping (Jagtap et al., 2016). SGCN is a non-metallic polymeric material with a narrow band gap (2.68 eV) to boost the stability and activity of the photocatalyst. Co_3_O_4_/g-C_3_N_4_ (GCO_2_) photocatalysts were reported by Vignesh et al. for the efficient removal of MO and MB (Suganthi and et a l., 2023).

The key goal of this work is to synthesize cobalt oxide (Co_3_O_4_), cadmium doped cobalt oxide (Cd-Co_3_O_4_) and their composites with SGCN (Cd-Co_3_O_4_@SGCN) as photocatalyst via the coprecipitation method to address the issue of the ever-growing dye pollution in textile industry wastewater by the photodegradation of Reactive Yellow 18. The novelty of this work is the fabrication of a Cd doped cobalt oxide@SGCN composite with high multifunctional environmental application. The synergistic combination of Cd doping and SGCN is an effective approach for tailoring the electronic structure, enhancing visible-light harvesting and promoting the charge migration at the interface. The composite photocatalyst exhibits the synergistic effect of photocatalytic degradation of RY-18 dye and antibacterial function, which shows good application potential in wastewater treatment and environmental remediation fields. Additionally, the composite’s outstanding recyclability underscores its structural reliability and usability. The synthesized NPs and NCs were used to estimate the antibacterial activity against two bacterial strains (*E.Coli*. and *B. Subtilis*).

## 2 Experimental

### 2.1 Precursors

Cobalt Nitrate (Co (NO_3_)_2_·6H_2_O), ammonium oxalate (97% purity), cadmium nitrate tetrahydrate (Cd (NO_3_)_2_·4H_2_O), thiourea (CH_4_N_2_S), reactive yellow 18 (C_25_H_16_ClN_9_Na_4_O_13_S_4_), ammonia, sodium hydroxide, Ethanol and hydrochloric acid were procured from Sigma-Aldrich and deionized water was used as a solvent. For the synthesis of nanomaterials, these chemicals were exploited without any purification.

### 2.2 Synthesis of cobalt oxide (Co_3_O_4)_

Cobalt oxide was synthesized by using a chemical precipitation process. To prepare cobalt oxide, 0.01 M cobalt nitrate hexahydrate solution was prepared in 50 mL of deionized H_2_O and a 0.01 M ammonium oxalate solution was added in it dropwise with constant stirring until pink precipitates of cobalt oxalate were obtained. Ammonia was used to maintain the pH at 5. Precipitates were filtered, washed with ethanol and deionized water (for the removal of nitrate ions and other impurities), dried (50 °C) for 2 hours and then calcinated (400 °C) for 4 hours. Black color cobalt oxide was obtained. To get powder, the synthesized cobalt oxide was crushed using a mortar and pestle.

### 2.3 Synthesis of cadmium doped cobalt oxide (Cd-Co_3_O_4_)

Cadmium doped cobalt oxide (1.3, 3.3, 5.3, 7.3 and 9.3 mol%) was synthesized by a chemical co-precipitation process. To synthesize 1.3% Cd-Co_3_O_4_, 1.3 mol% of cadmium nitrate tetrahydrate and 0.01 M cobalt nitrate hexahydrate were prepared in 50 mL of deionized water separately by continuous stirring. Cadmium solution was added to the cobalt nitrate solution with constant stirring for 30 min to obtain a homogenized solution. 0.01 M ammonium oxalate solution was added dropwise into it with constant stirring until pink color cadmium-doped cobalt oxalate precipitates were obtained. pH of the solution was maintained at 5 by adding ammonia dropwise. Precipitates were filtered, washed (with deionized H_2_O and ethanol for removing nitrate ions and other impurities), dried (50 °C) for 2 hours and then calcinated (400 °C) for 4 hours resulting in the formation of black color cadmium doped cobalt oxide nanoparticles. The obtained material of cadmium doped cobalt oxide was then crushed using a mortar and pestle to get powder.

### 2.4 Synthesis of SGCN

SGCN was prepared from thiourea via employing a calcination process. A measured quantity of thiourea was placed in crucibles, which were positioned inside a furnace with an air atmosphere and the beginning temperature of 25 °C. Gradually the temperature was elevated to 500 °C and then the sample was kept there for 3 hours. After cooling the resultant yellowish product was crushed to get powder.

### 2.5 Synthesis of Cd-Co_3_O_4_@SGCN nanocomposites

**FIGURE 1:**
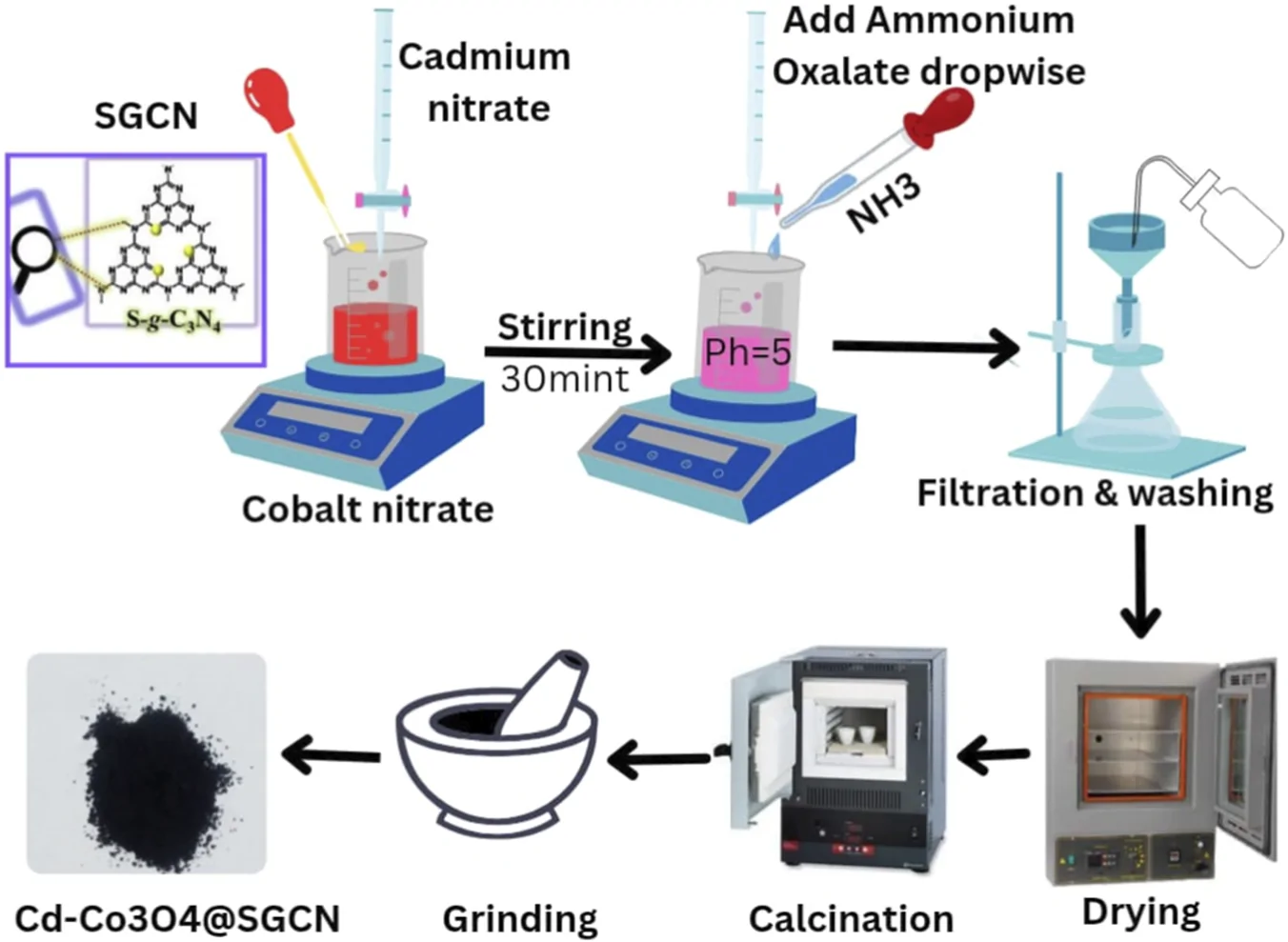
Pictorial illustration for synthesis of the Cd-Co_3_O_4_@SGCN nanocomposite.

By varying SGCN percentage (10, 30, 50 and 70 mol%), 5.3% cadmium doped cobalt oxide nanocomposites with SGCN were prepared using a chemical co-precipitation. To synthesize 5.3% Cd-Co_3_O_4_@10%SGCN 0.1887 g of SGCN, 5.3 mol% of cadmium nitrate tetrahydrate and 0.01 M cobalt nitrate hexahydrate were prepared in 50 mL of deionized H_2_O separately on continuous stirring and poured both solutions (cadmium and SGCN) to the cobalt nitrate solution with constant stirring (30 min) to obtain a homogenized solution. Then 0.01 M ammonium oxalate was added with constant stirring until pink color oxalate precipitates were obtained and ammonia was used to keep the pH at 5. Precipitates were filtered, washed with ethanol and deionized water to reduce nitrate ions and other impurities, dried (50 °C) for 2 hours and then calcinated at 400 °C for 4 hours, resulting in the formation of black color cadmium doped cobalt oxide nanocomposites. The synthesized 5.3% cadmium doped cobalt oxide nanocomposites were crushed by a mortar and pestle to get powder. For the ease of understanding Figure 1 provides a Pictorial illustration for the synthesis of Cd-Co_3_O_4_@SGCN nanocomposite.

### 2.6 Characterization of synthesized nanoparticles

The synthesized Co_3_O_4_, Cd-Co_3_O_4_ NPs and Cd-Co_3_O_4_@50% SGCN NCs were characterized using XRD, FTIR, SEM, EDX and UV-VIS spectroscopy. The crystallinity of NPs and NCs was confirmed using a Bruker D2-diffractometer that works at 10 mA, 30 kV by using a copper tube with a wavelength of 0.15418 nm and 2θ with a range of 10°–70°. The functional group study was obtained by FTIR analysis using an FTIR spectrometer operating in the 4000-400 cm^-1^ range. EDX for elemental analysis was employed in conjunction with a SEM (SEM-JEOL, IT 200 Japan) to ascertain the morphology and surface properties of the materials. An ultraviolet-visible spectrophotometer with a wavelength range of 200–800 nm was used to measure the samples photocatalytic degradation efficacy.

### 2.7 Photocatalytic activity

To determine the optimal photocatalytic behavior of the synthesized NPs and NCs, RY-18 (organic pollutant) was used. Photocatalytic behavior of Co_3_O_4_, Cd-Co_3_O_4_ and Cd- Co_3_O_4_@SGCN was studied under UV-Visible spectroscopy with the aid of RY-18 dye degradation at a wavelength (λ) of 410 nm. For this purpose, 0.1 g of photocatalyst was added to 100 mL of RY-18 solution and sonicated for 15 min to attain absorption-desorption equilibrium between the catalyst surface and the given pollutant (Shanaah et al., 2024). After that, solutions according to various concentrations were placed under sunlight until the solutions degraded completely. From that sample, a 5 mL aliquot of RY-18 solution was taken every 30 min which was used to analyze photocatalytic activity by UV-vis spectrum.

### 2.8 Antibacterial activity

The antibacterial properties of Co_3_O_4_, Cd-Co_3_O_4_ and their composites SGCN were investigated against *E. coli*. and *B. subtilis* strains by agar well diffusion method.

#### 2.8.1 Preparation of inoculum

Inoculums for the negative and positive bacterial strains was produced independently by dissolving 0.64 g of nutritional broth in 50 mL of deionized water and then stir it for 15 min. Flasks were removed from the autoclave after 20 min and allowed to cool at room temperature. Two to three drops of strain were added to this solution. After that, both flasks were left in the shaker for 24 hours.

#### 2.8.2 Preparation of petri plates

Petri dishes were autoclave-sterilized and then permanently marked into four sections. Three holes for sample solutions with different concentrations were included in these sections, along with one blank hole (Javed et al., 2023). A flask containing 200 mL of distilled water was then filled with 8 g of nutrient agar and 4 g of nutrient broth. After being autoclave sterilized, this mixture was boiled for five to ten minutes. Petri dishes that had already been prepared and stored in a laminar flow cabinet were filled with the mixture. The inoculum was applied to the gel’s surface after the mixture had cooled. A borer was used to drill tiny wells in each section once it was totally dry. Using a micropipette for standard solutions with different concentrations, i.e., 200 ppm, 250 ppm, 300 ppm and 350 ppm were added to the wells. For twenty-four hours, every petri dish was kept in the incubator at 37 °C. Results were attained through calculating the zone of inhibition (ZOI) diameter and comparing it to the blank.

## 3 Results and discussion

### 3.1 FTIR analysis

The functional group and chemical composition of Co_3_O_4_, Cd-Co_3_O_4_ and their composites with SGCN were characterized by FTIR spectroscopy (Alharbi, 2024). Distinct peaks of single or mixed oxides in the 1,000 to 400 cm^-1^ range are known to result from the vibration of metallic ions within a crystal’s structure (Al-Senani et al., 2020). The peaks for the Co^2+^ functional group were observed at about 662, 659 and 647 cm^-1^ for Co_3_O_4_, Cd-Co_3_O_4_ and 5.3% Cd-Co_3_O_4_@SGCN respectively, which confirms the presence of cobalt oxide. In a similar pattern, peaks for Co^3+^ were observed at 559,554 and 548 cm^-1^ (Bahadoran et al., 2022; Prabaharan et al., 2017). Hydroxyl stretching vibration is responsible for the peak in the range of 3,300–3,400 cm^-1,^ which was most likely caused by moisture adsorption from the environment. At 1,600–1700 cm^-1,^ another band is seen due to the presence of the C=O vibrational peak (Asha et al., 2022; Jeyachitra et al., 2018; Reena et al., 2022).

**FIGURE 2:**
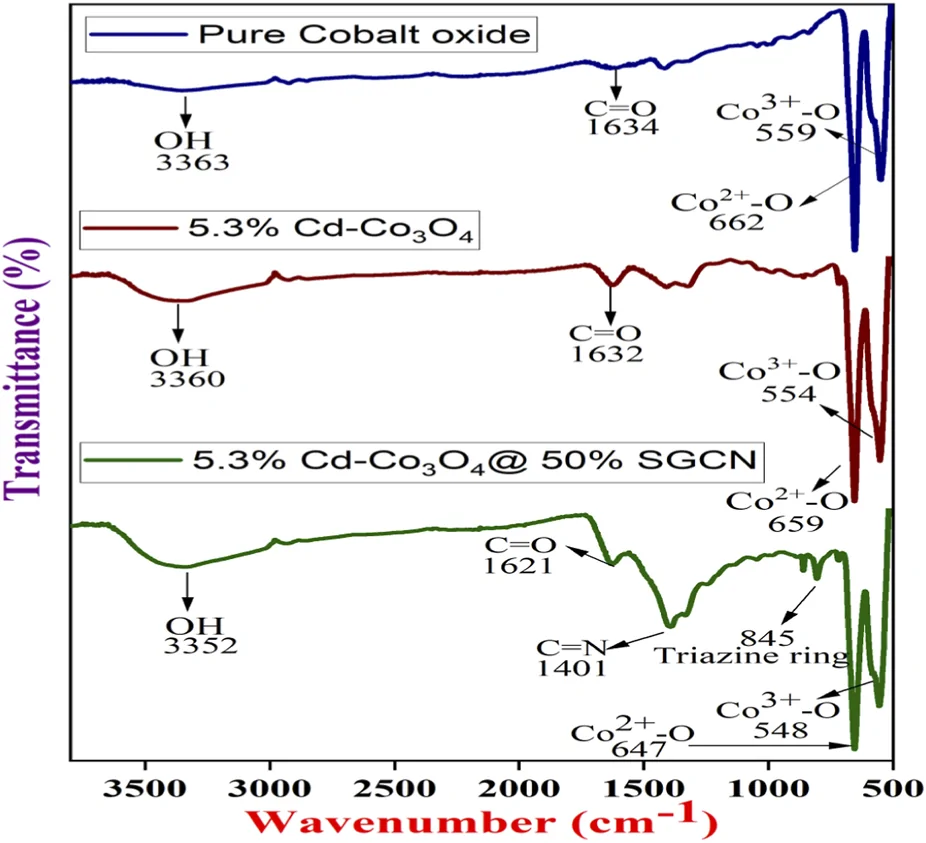
FTIR spectra of Co_3_O_4_, 5.3% Cd-Co_3_O_4_, SGCN and 5.3% Cd-Co_3_O_4_@50% SGCN.

The shifting of Co^3+^-O peaks from 559 to 548 cm^-1^ and other peaks also moved towards lower wavenumber and higher wavelength. Additionally, the peaks position for Cd-Co_3_O_4_ nanocomposites slightly changed because of the small amount of SGCN. When Cd- Co_3_O_4_ was combined with SGCN, it evidently indicated a bathochromic shift which causes absorption in the visible region, verifying the successful synthesis of the compounds. FTIR spectra of pure cobalt oxide, 5.3% Cd-Co_3_O_4_ and 5.3% Cd-Co_3_O_4_@50% SGCN are represented in Figure 2.

### 3.2 X-ray diffraction (XRD) analysis

**FIGURE 3:**
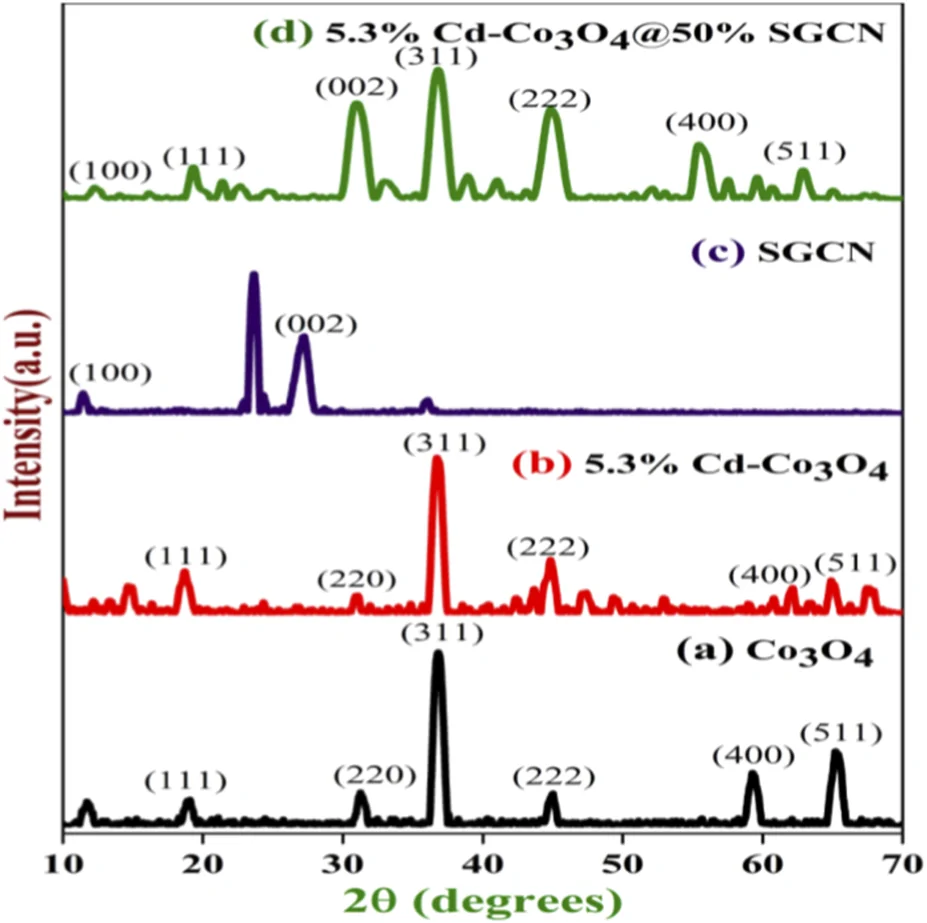
XRD pattern of **(a)** Co_3_O_4_, **(b)** 5.3% Cd-Co_3_O_4_, **(c)** SGCN and **(d)** 5.3% Cd-Co_3_O_4_ @50% SGCN.

Crystalline phases were identified by XRD spectroscopy. XRD of synthesized NPs and NCs was executed at 10°–70°. The obtained pattern of cobalt oxide demonstrated that there were no new impurity peaks in the pattern. The Co_3_O_4_ nanoparticle diffraction peaks (Figures 3a,b) at 2ϴ (19.00, 31.008, 36.88, 44.72, 59.18, and 65.19) are caused by the (111), (220), (311), (222), (400), and (511) planes. The obtained peaks of Co_3_O_4_ nanoparticles align perfectly with the standard JCPDS card numbers 042–1,467 and 74–2,102, confirming its cubic spinel structure (Bibi et al., 2017; Khan et al., 2023; Pallavolu et al., 2022). X-ray diffraction pattern for cadmium doped cobalt oxide demonstrates that the pattern closely resembles the XRD spectra of pure cobalt oxide, with a slight shift in the peaks and a decrease in intensity. Nonetheless, the grain size and lattice parameter of cobalt are reduced by doping. Figure 3c displays the XRD pattern for SGCN.

In this pattern two distinct bands can be seen at 12.09° and 27.2°, which represent the 100 and 002 facets of the crystal respectively. JCPDS number 00-087-1526 provides documentation of the successful synthesis of SGCN (Ghafuri et al., 2021; Javed et al., 2020; Khan et al., 2023). The XRD pattern of 5.3% Cd-Co_3_O_4_ @50% SGCN shows several distinct peaks. The existence of sulfur-doped GCN in the 002 facet is responsible for the major peak at 27.4° and the minor peak at 14°. The additional peaks located at (111), (220), (311), (222), (400) and (511) confirm the existence of Cd-doped Co_3_O_4_. Peak intensity obtained from the XRD pattern of 5.3% Cd-Co_3_O_4_@50% SGCN was less than that of Co3O4 and 5.3% cadmium-doped Co_3_O_4_. Additionally, the successful synthesis of 5.3% cadmium doped cobalt oxide nanocomposites with 50% SGCN is confirmed by a slight shift to lower theta with peak broadening. These findings reveal that SGCN insertion in cadmium doped cobalt oxide nanoparticles reduced the grain size, which provides a large surface area and high porosity. XRD pattern of Co_3_O_4_, 5.3%Cd-Co_3_O_4_, SGCN and 5.3%Cd-Co_3_O_4_@50%SGCN is revealed in Figure 3. Structural properties including average crystallite size, microstrains and dislocation density were calculated from XRD data and given in Table 1. The average crystallite sizes for Co_3_O_4_, 5.3%Cd-Co_3_O_4_ and 5.3%Cd-Co_3_O_4_@SGCN were determined to be 14, 12 and 9 nm, respectively (Kerangueven et al., 2022).

**TABLE 1:** Structural properties calculated from XRD data.

Sample	Peak position 2θ(degree)	FWHM(β) degrees	Average crystallite size	Dislocation density δ = 1/D^2^ × 10^3^nm^-2^	ϵ = β/4Tanθ
Co_3_O_4_	36.81091	0.72109	13.32297	0.005711	0.443946
5.3% Cd- Co_3_O_4_	36.63286	0.74090	12.24817	0.005276	0.73254
5.3% Cd-Co_3_O_4_@50%SGCN	35.07596	1.26377	9.379621	0.007418	1.046984

### 3.3 Morphological analysis

**FIGURE 4:**
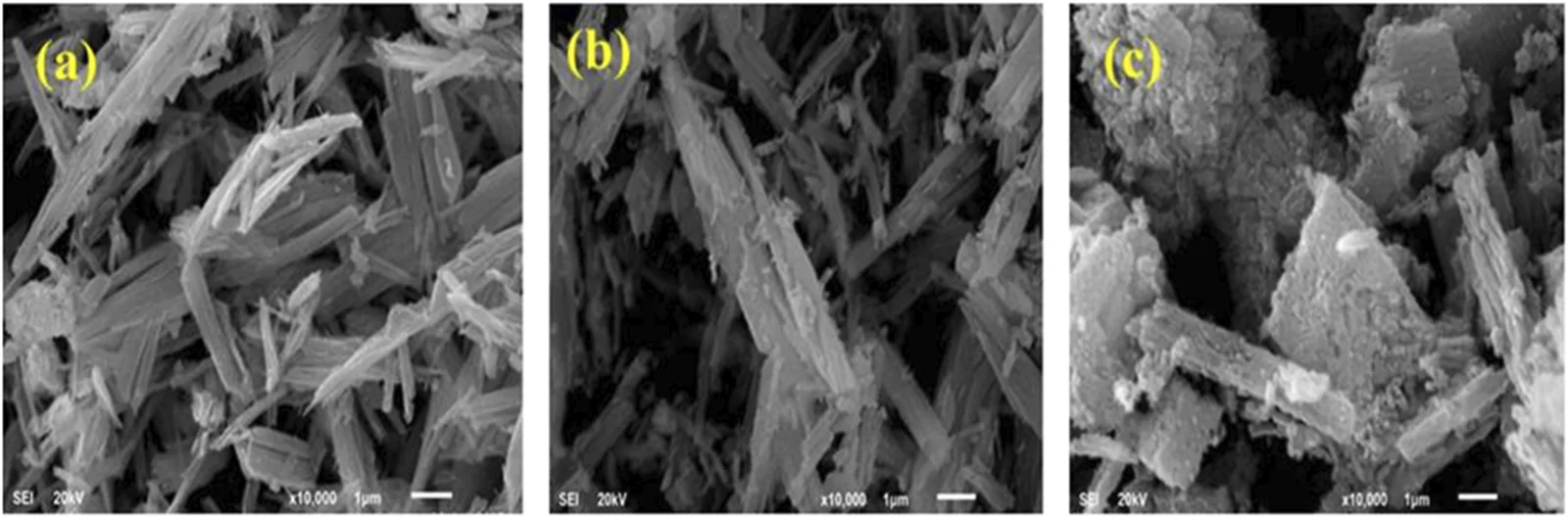
SEM images of **(a)** Co_3_O_4_, **(b)** 5.3%Cd-Co_3_O_4_ NPs and **(c)** 5.3%Cd-Co_3_O_4_@SGCN NCs.

The morphology of the pure Co_3_O_4_, Cd-Co_3_O_4_ and Cd-Co_3_O_4_@SGCN was observed using SEM. Figure 4 displays the observed SEM pictures of nanostructures at a scale of 1 μm and 20 kX magnification. Figures 4a,b clearly depicts an agglomerated 1D nanorod-like structure with a uniform diameter of the pure Co_3_O_4_ and Cd-Co_3_O_4_ (Durano et al., 2017; Rani et al., 2017). The rod and sheet-like morphologies were observed for Cd-Co_3_O_4_@SGCN in Figure 3c. The even distribution of the nanosheets suggests a controlled synthesis process, where the particles were well dispersed by continuous stirring during synthesis. On the surface of NCs, agglomeration and aggregation of NPs were also observed.

### 3.4 Elemental analysis

**FIGURE 5:**
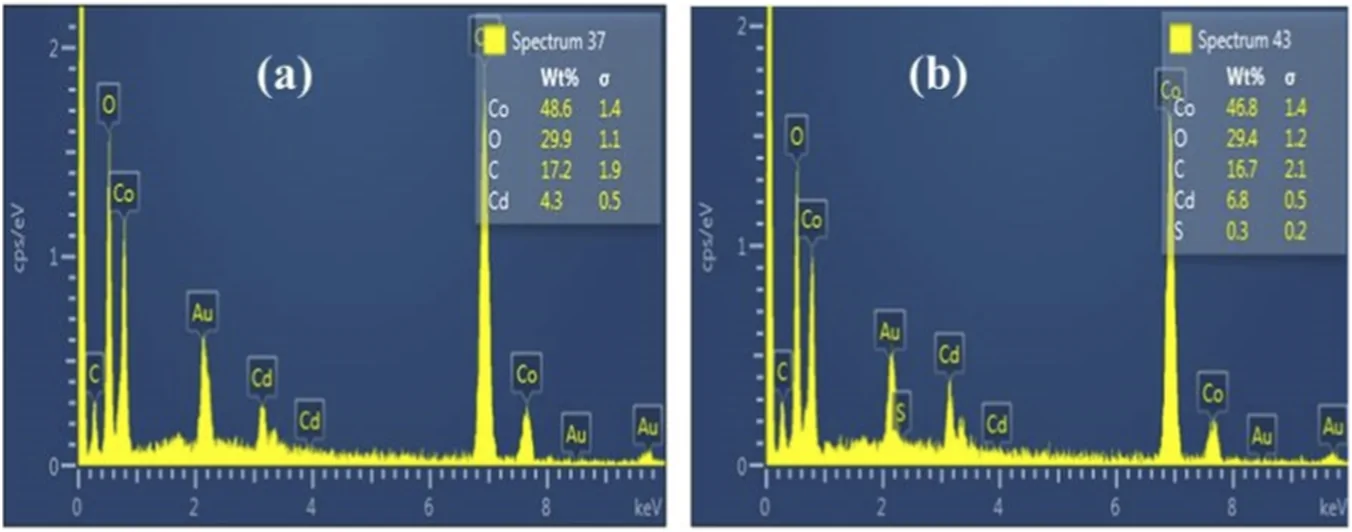
EDX images of **(a)** Cd-Co_3_O_4_ NPs and **(b)** Cd-Co_3_O_4_@SGCN NCs.

Using EDX spectroscopy, the elemental composition of Co_3_O_4_, Cd-Co_3_O_4_ and Cd-Co_3_O_4_ nanocomposites with SGCN was examined, as illustrated in Figures 4a,b. Using an internal standard, the EDX analysis of the NPs and NCs was performed at an energy of 0–10 keV. The Co_3_O_4_ and Cd-Co_3_O_4_ (Figure 5a) spectra showed the existence of cobalt (Co), cadmium (Cd), oxygen (O), carbon (C) and elements with slightly higher atomic and weight % of cobalt. Likewise, the Cd-Co_3_O_4_ @SGCN spectrum (Figure 5b) reveals the presence of cobalt (Co), cadmium (Cd), oxygen (O), carbon (C) and sulfur (S) elements. The weight percentages of these elements are also given, in addition to the elemental analysis. The elements identified in the spectrum validate the effective fabrication of the Cd-Co_3_O_4_ nanocomposite with SGCN.

### 3.5 Optical properties

The optical behavior and bandgap of all the prepared nanoparticles and nanocomposites were measured at room temperature using UV-visible spectroscopy. By using the following Equations 1–3, the band gap of the prepared samples was measured.

E=hv=1240λ (1)

$$\alpha =4\pi \left(Absorbance\right)/\lambda$$ (2)

$$\left(\alpha hv\right)=B{\left(hv-H_{g}\right)}^{1/2}$$ (3)

**FIGURE 6:**
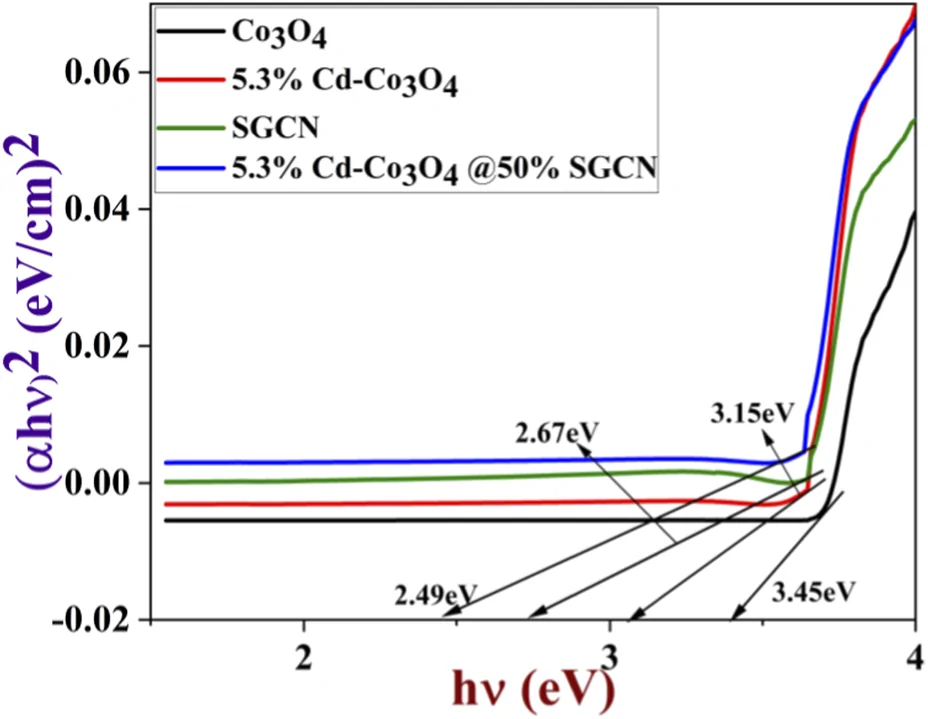
Bandgap of Co_3_O_4_, 5.3% Cd-Co_3_O_4_, SGCN and 5.3% Cd-Co_3_O_4_@50% SGCN.

The band gap is the most important property for assessing the catalytic efficiency of the synthesized nanomaterial. By using Tauc’s plot band gap was calculated for pure cobalt oxide, Cd-Co_3_O_4_ and Cd-Co_3_O_4_@SGCN as depicted in Figure 6. The calculated band gap for Co_3_O_4_ was approximately 3.45 eV (Farhadi et al., 2013; Vennela et al., 2019) That is very close to the reported value, which decreases to 3.15 eV on doping with Cd, confirming the significant decrease in the e^−^ and h^+^ pair recombination rate and raising the photodegradation efficiency of RY-18. For SGCN, the estimated band gap is 2.67 eV. SGCN bandgap is comparable to that which has been earlier documented (Ramzan et al., 2023). This hybridization of SGCN with Cd-Co_3_O_4_ caused a further reduction of the band gap to 2.49 eV. From the results, it was concluded that the formation of composites improves the photodegradation efficiency of Cd-Co_3_O_4_ NPs.

### 3.6 Photocatalytic degradation activity by synthesized NPs and NCs

#### 3.6.1 Mechanism of photocatalytic degradation

**FIGURE 7:**
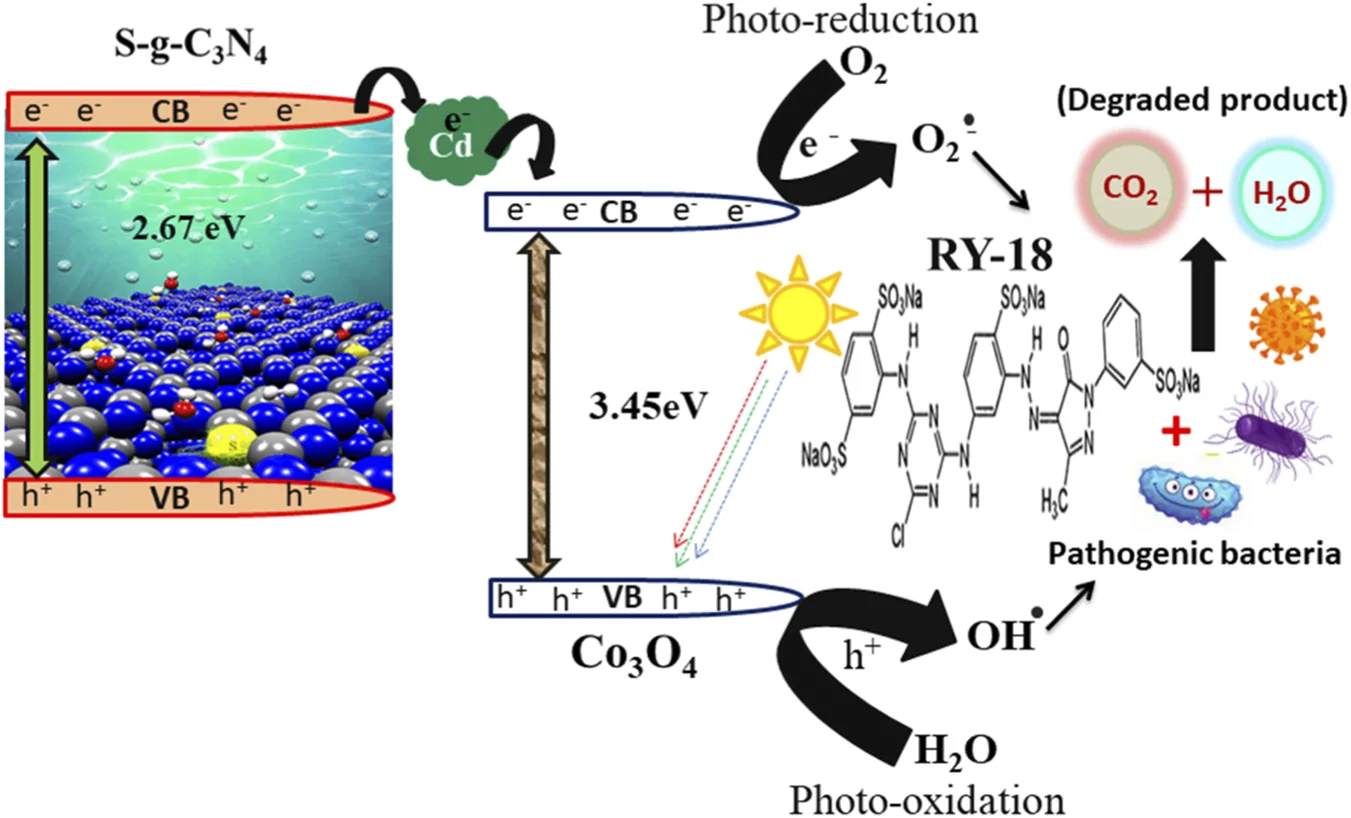
Photocatalytic RY-18 degradation mechanism over the Cd-Co_3_O_4_@SGCN.

According to a schematic diagram, the immediate degradation of dye and antibacterial activity of photocatalysts may be due to the formation of e^−^/h^+^ pairs in the catalysts. The quick breakdown of reactive yellow 18 and the antibacterial properties of photocatalysts are ascribed to Cd-Co_3_O_4_/S-g-C_3_N_4_ (Figure 7). Cd-Co_3_O_4_ and SGCN are both excited, electron and hole pairs are photogenerated on their VB and CB respectively, when exposed to solar radiation. The photo-induced electrons easily moved between the two bands because the conduction band (CB) of Cd-Co_3_O_4_ had a lower potential than the CB of S-g-C_3_N_4_ based on the energy level of CB/VB. Furthermore, S-g-C_3_N_4_ captures the holes that were formed in the VB of Cd-Co_3_O_4_ (Sher et al., 2021). In addition to lowering the value of the bandgap (Eg), the hybrid composites of Cd atoms made it easier for electrons to move from S-g-C_3_N_4_ to Cd-Co_3_O_4_. The addition of SGCN not only enhances visible light absorption but also enables the transfer of charge between SGCN and Cd-Co_3_O_4_. The extended π-conjugated structure of SGCN provides an efficient pathway for photogenerated charge migration, which promotes spatial separation of electrons and holes and suppresses their recombination. Therefore, the long-life of charge carriers increases the generation of ROS species participating in photocatalytic reactions, which leads to better photocatalytic performance. Doping may thus greatly reduce the likelihood of charge recombination by increasing the spacing of photoexcited e^−^/h^+^ couples. Reactive oxygen species (ROS) are created when the photogenerated e^−^/h^+^ couples interact with water and oxygen molecules on the photocatalyst’s surface. After that, the generated radicals oxidatively destroy pathogenic bacteria and degrade RY-18 dye (Zhong et al., 2020).

#### 3.6.2 Photocatalysis by Co_3_O_4_, Cd-Co_3_O_4_ NPs and Cd-Co_3_O_4_@SGCN

The ability of Co_3_O_4_, Cd-Co_3_O_4_ NPs and Cd-Co_3_O_4_@SGCN to degrade a reactive azo dye (RY-18) when exposed to sunlight was evaluated using a UV-visible spectrophotometer, which measures wavelengths between 200 and 800 nm and a model dye (RY-18). The absorbance of RY-18 for each of the doped NPs decreased with time, according to the UV-visible spectra, suggesting a rise in RY-18 degradation. The following formula (Equation 4) was used to determine the rate of degradation for reactive yellow 18 at different concentrations of synthesized nanoparticles and nanocomposites.

Rate of degradation=C/CO (4)

**FIGURE 8:**
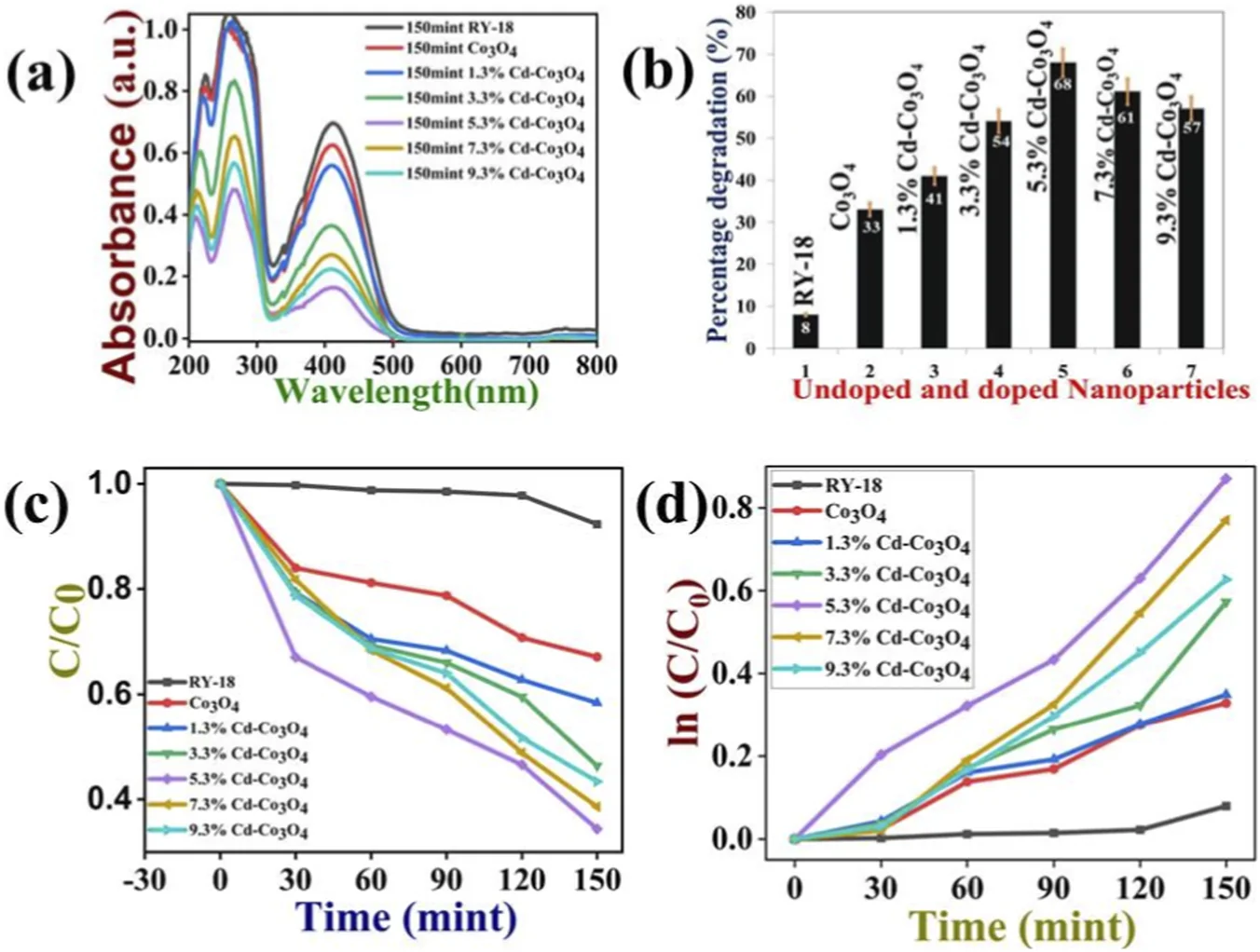
Degradation results of NPs: **(a)** Absorption curves of photodegradation of RY-18, **(b)** degradation efficiency, **(c)** rate of degradation and **(d)** plot of ln (Co/C) by Co_3_O_4_ and Cd-Co_3_O_4_.

Figures 8a,b represents that, in comparison to other synthesized samples, 5.3% Cd-Co_3_O_4_ NPs demonstrated the maximum degradation of RY-18 (68% in 150 min). The photocatalytic efficiency of Co_3_O_4_ and (1.3, 3.3, 5.3, 7.3 and 9.3 weight percent) Cd-Co_3_O_4_ NPs against RY-18 is displayed in Figure 8b. Pure Co_3_O_4_ degraded less dye than Cd-Co_3_O_4_ nanoparticles, according to Figure 8c plots of C/C_o_ versus time and Figure 8d shows the kinetic study for the degradation of RY-18 in the presence of photocatalysts. To enhance photocatalytic degradation 5.3% cadmium doped cobalt oxide introduced defects level between the conduction and valence bands due to oxygen states and Cd ions, which behave as trapping centers for hole and electron pairs. For increasing photocatalytic action, these trapping centers reduce the electron-hole pair recombination. However, photocatalytic efficiency increased at the Cd doping of 5.3% after which it began to decrease. In addition to providing more centers for electron-hole pair recombination, a high level of doping lowers the photocatalytic action (Sowjanya et al., 2020). The photocatalytic degradation efficiency of Cd-Co_3_O_4_ gradually increased with the increase of Cd concentration from 1.3% to 5.3%, and the degradation efficiencies were 41%, 54% and 68%, respectively. This improvement is attributed to band gap modification and enhanced charge carrier generation. This efficiency was however reduced by adding more Cd (7.3% and 9.3%) which led to greater electron–hole recombination caused by the excessive amounts of dopants. Thus, it is concluded that the optimum amount of Cd doping for better photocatalytic activity is 5.3%.

**FIGURE 9:**
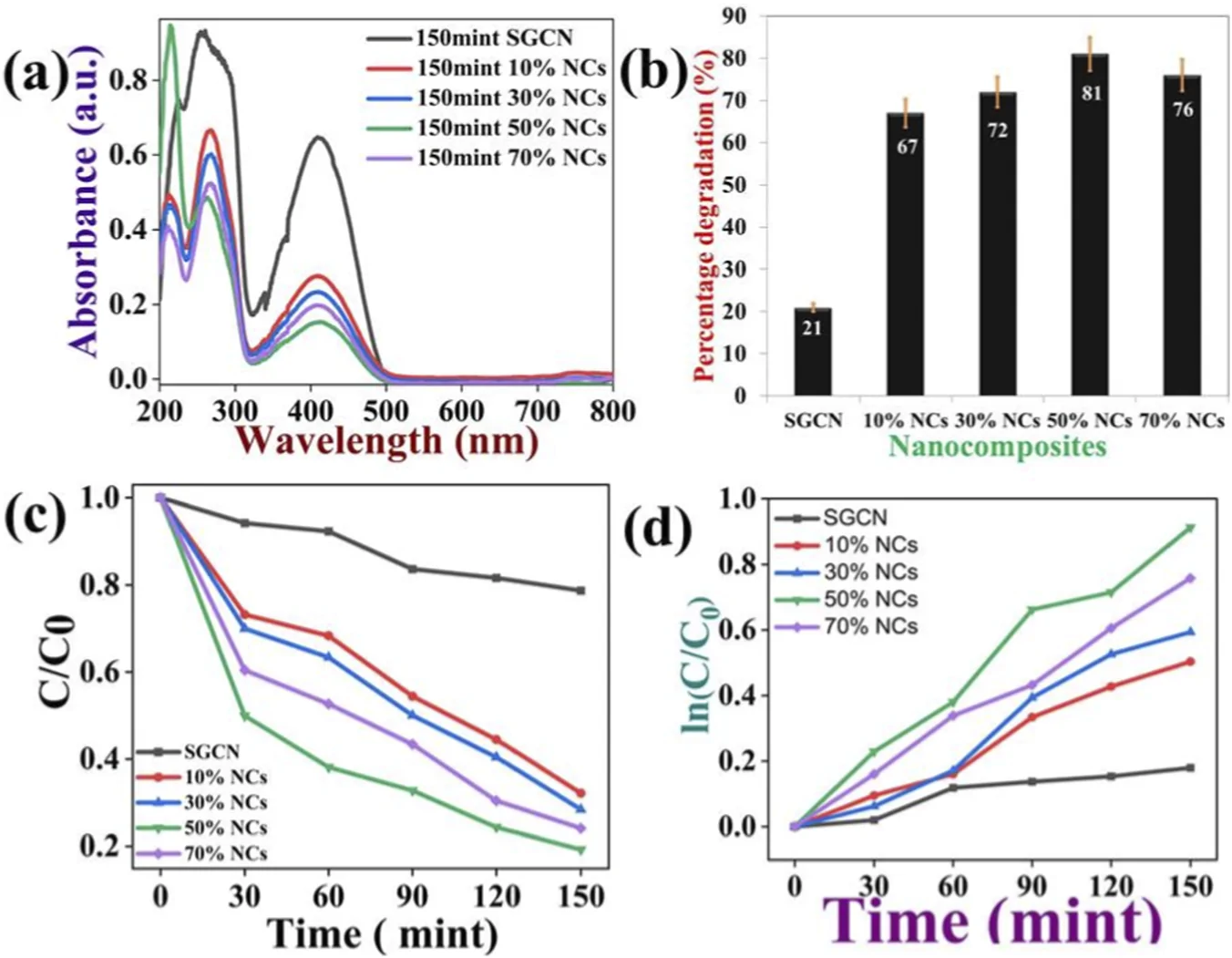
Degradation results of NPs: **(a)** Absorption curves of photodegradation of RY-18 solutions, **(b)** degradation efficiency, **(c)** rate and **(d)** plot of ln (Co/C) by SGCN and Cd-Co_3_O_4_@SGCN.

As compared to Co_3_O_4_ and Cd-Co_3_O_4_ nanoparticles, Cd-Co_3_O_4_@SGCN showed superior absorption, resulting in a higher rate of degradation. By varying the concentration of SGCN (10, 30, 50% and 70%) Cd-Co_3_O_4_@SGCN were prepared to check their photodegradation efficiency. Degradation efficiency gradually increases by increasing the concentration of SGCN up to 50% then decreases (67, 72, 81% and 76%) due to electron hole pair regeneration. Among all the samples, the Cd-Co_3_O_4_@50% SGCN exhibited the maximum degradation; it degraded 81% of the RY-18 dye in 150 min at pH 7, as depicted in Figure 9. Due to the establishment of an effective heterojunction between Cd-Co_3_O_4_ and SGCN, this inhibits e^−^ and h^+^ pair recombination and boosts the photodegradation of RY-18. The strong synergistic interaction between SGCN and Cd-Co_3_O_4_ offers more defects in the heterostructures giving the dye more places to bind, which makes the dye more prone to degradation. Table 2 compares the degradation efficiency of photocatalysts with previous work.

**TABLE 2:** Comparison of degradation efficiency utilizing Cd-Co_3_O_4,_ Cd-Co_3_O_4_@SGCN.

Photocatalyst	Dye	Source of radiation	Time (mint)	Degradation (%)	References
Co NPs	MB	Sunlight	300	88	Vinayagam et al. (2023)
Tragacanth gum based Cr-MnO_2_/clay NCs	RY-18	solar	120	93	Elahi et al. (2025)
CoO NPs	TY	Hg lamp	40	51	Khan et al. (2019)
Co_3_O_4_/g-C_3_N_4_	MO	Solar	210	65	Suganthi and et a l. (2023)
Cd-Co_3_O_4_	RY-18	Solar	150	68	This study
Cd-Co_3_O_4_@50%SGCN	RY-18	Solar	150	83	This study

### 3.7 Stability of photocatalyst

**FIGURE 10:**
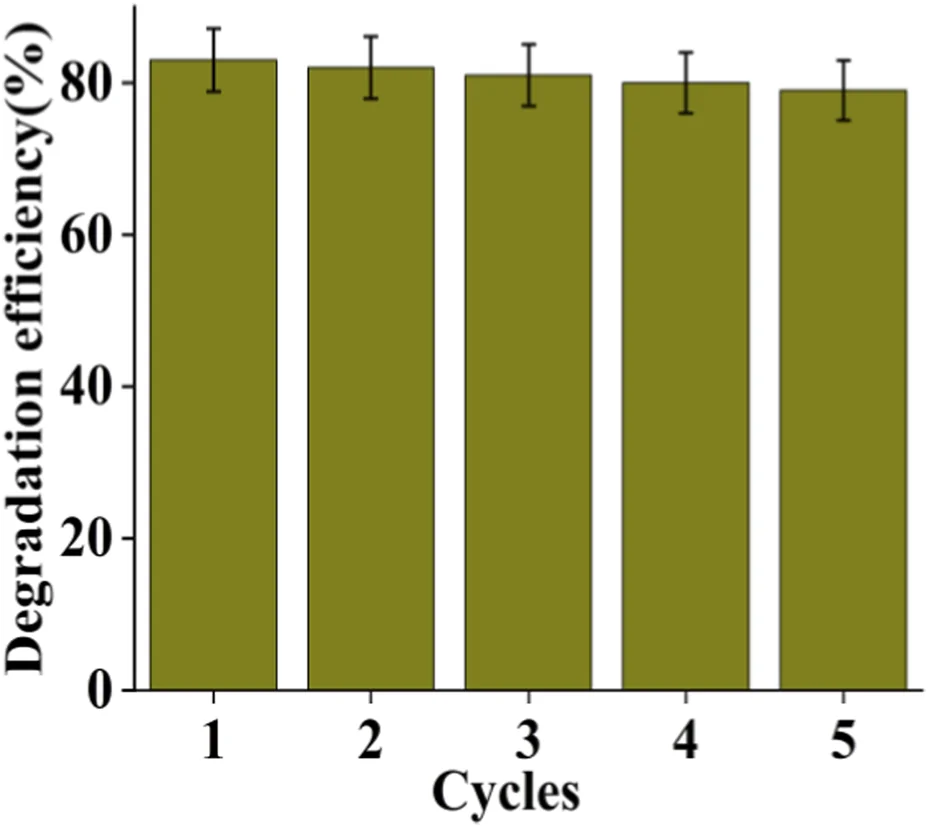
Stability and reusability of Cd-Co_3_O_4_@SGCN by degradation efficiency.

Five cycles of photocatalytic degradation measurements of RY-18 were carried out to perform further investigation on the stability and recyclability of the Cd-Co_3_O_4_@SGCN heterojunction (Figure 10). Degradation efficiency of photocatalyst dropped by just 1% after five cycles demonstrating the Cd-Co_3_O_4_@SGCN heterojunctions exceptional structural stability and reusability.

### 3.8 Effect of pH on photodegradation of RY-18

**FIGURE 11:**
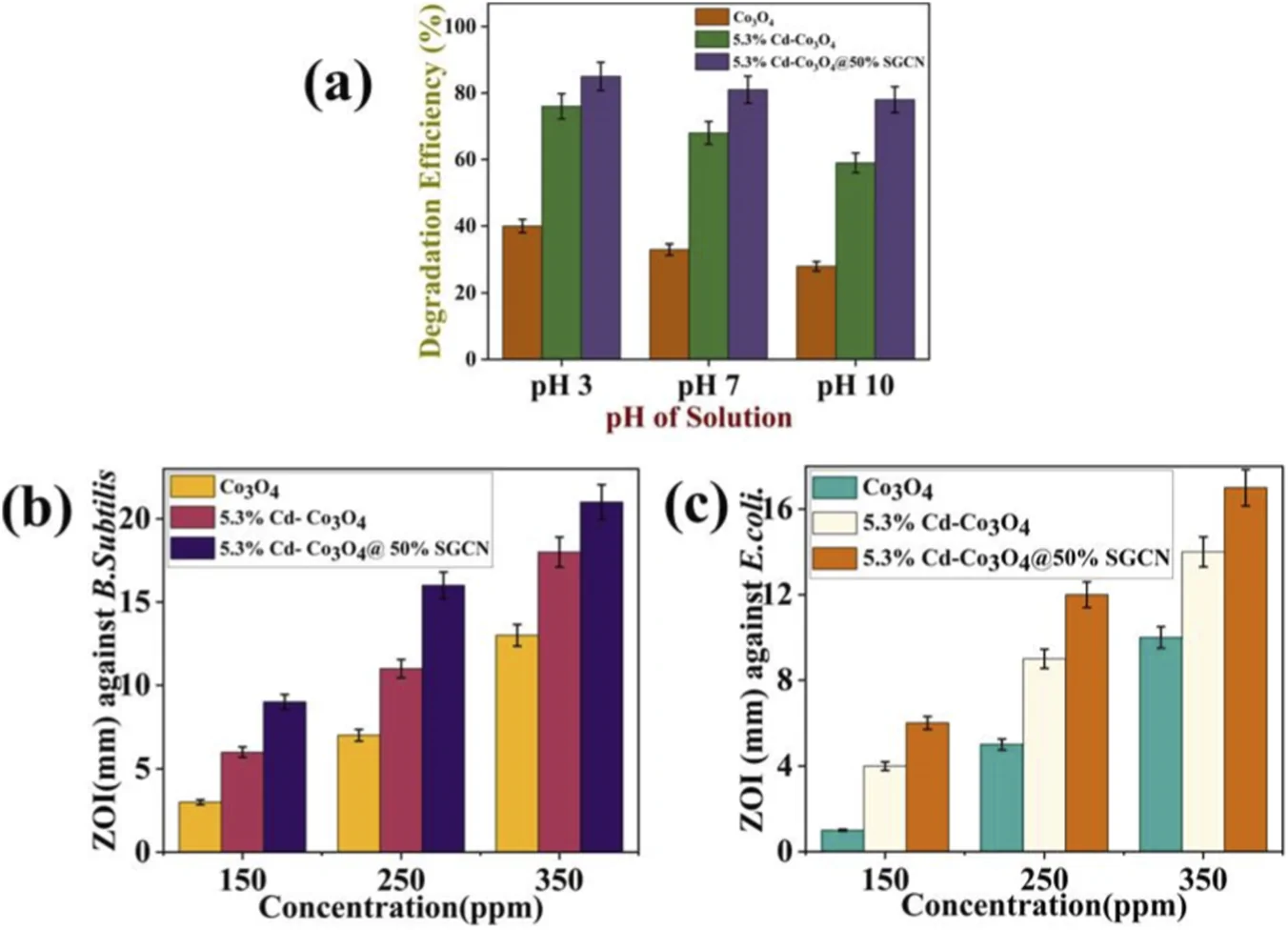
**(a)** Effect of pH on photocatalytic degradation of RY-18, **(b)** ZOI against *B. Subtilis* and **(c)** ZOI against *E. coli.* by Co_3_O_4_, 5.3% Cd-Co_3_O_4_ and 5.3%Cd-Co_3_O_4_@50%SGCN.

pH of the solution is an important factor for the photocatalytic reaction. It significantly affects the adsorption of dyes at the photocatalyst surface. The pH of reactive yellow 18 solutions was maintained by adding NaOH or HCl (1 M). In the range of 3–10, the impact of solution pH on the rate of photodegradation was examined while maintaining a constant dye concentration and catalyst amount of 0.1 g/100 mL. It was noticed that the removal of dye by Co_3_O_4_, Cd-Co_3_O_4_ and Cd-Co_3_O_4_@SGCN was slow at first and then accelerated as contact time increased. A decrease in the degradation of RY-18 dye was observed with increasing the pH from 3 to 10. From the results, it was concluded that 5.3% Cd-Co_3_O_4_@50%SGCN nanomaterial exhibited optimal degradation at pH 3 then Co_3_O_4_ and Cd-Co_3_O_4_ as revealed in Figure 11a.

### 3.9 Antibacterial activity

**FIGURE 12:**
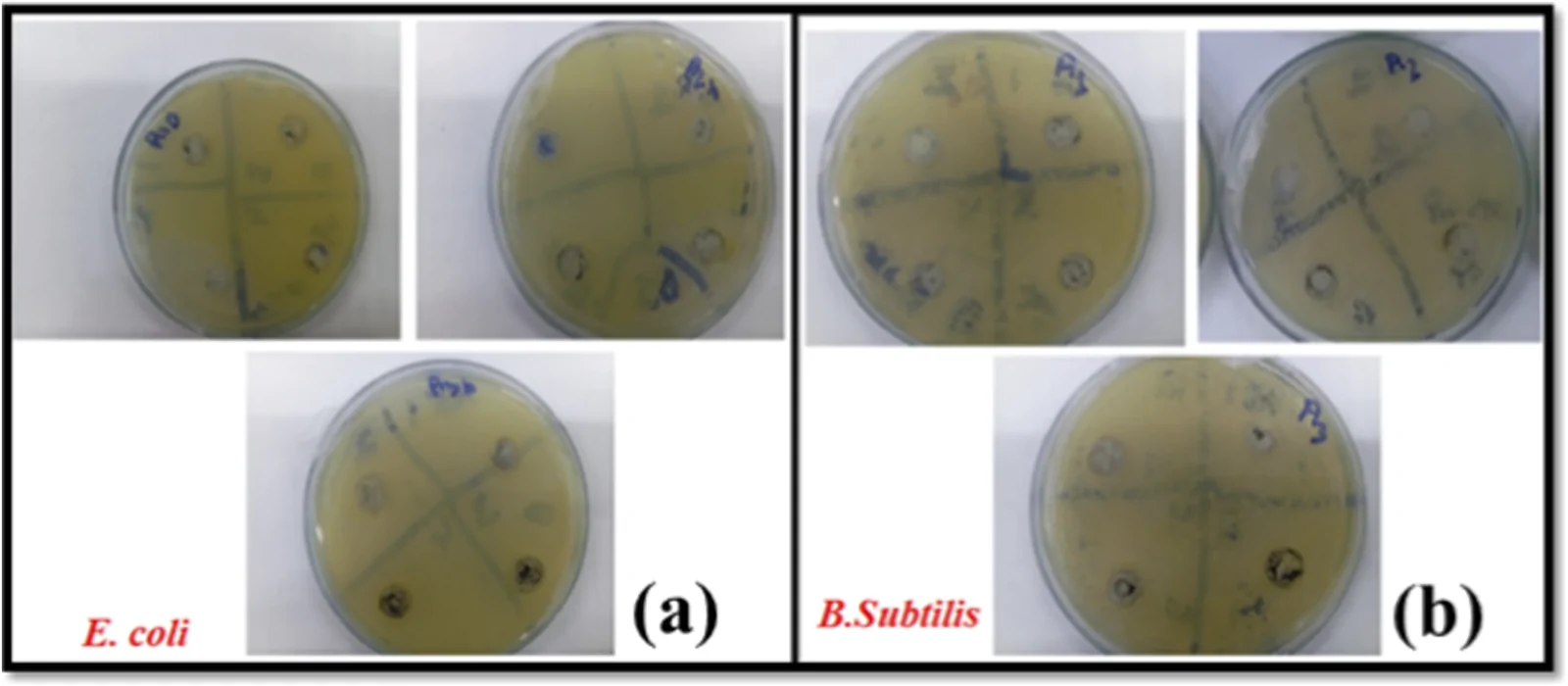
Antibacterial activity against **(a)** *E. coli*. and **(b)** *B. Subtilis*.

Using different concentrations of Co_3_O_4_, Cd-Co_3_O_4_ and Cd-Co_3_O_4_@SGCN (150, 250 and 350 mg/L solutions), the antibacterial activity was investigated against two bacterial strains (*B. subtilis* and *E. coli*.). By measuring the zone of inhibition (ZOI), the corresponding bactericidal results were recorded and are provided in Figures 11b,c. A concentration of 350 mg/L of Cd-Co_3_O_4_/50% SGCN was found to exhibit outstanding antibacterial activity, producing a 17 ± 0.2 mm ZOI against *E. coli* and 21 ± 0.2 mm ZOI against *B. subtilis* (Table 3; Figure 12). It was discovered that the samples’ ordered bactericidal effect on bacteria was Co_3_O_4_<Cd-Co_3_O_4_<Cd-Co_3_O_4_/SGCN. The prepared nanocatalysts produce reactive oxygen species (ROS) and e^–^/h^+^ pairs when exposed to light, in accordance with the suggested photocatalytic and bactericidal mechanism.

**TABLE 3:** Antibacterial activity of synthesized NPS and composites against *B. Subtilis* and *E. coli*.

Samples	Diameter of ZOI (mm) for different concentrations (ppm)					
	*B. subtilis*			*E. coli*		
Co_3_O_4_	3	6	9	1	4	6
Cd- Co_3_O_4_	7	11	16	5	9	12
Cd- Co_3_O_4_@SGCN	13	18	21	10	14	17

## 4 Conclusion

In this research work, cobalt oxide (Co_3_O_4_), Cd-Co_3_O_4_ (1.3%–9.3%) and Cd-Co_3_O_4_ nanocomposites with SGCN (10%–70%) were successfully synthesized as a photocatalyst to investigate the degradation of reactive yellow 18.5.3% Cd-Co_3_O_4_ demonstrates the efficient degradation of RY-18 (68%) after 150 min of exposure to sunlight. The XRD results confirm the cubic spinel structure of pure cobalt oxide and increasing the concentration of cadmium as a dopant decreased the crystalline nature of cobalt oxide. Band gaps of all the nanoparticles and nanocomposites were calculated from the Tauc’s plots and the band gap for 5.3% Cd-Co_3_O_4_@50%SGCN was 2.49 eV. It was concluded that dopant concentration can affect electron transport directly to reduce the band gap and boost the photodegradation of RY-18. It was observed that 5.3% Cd-Co_3_O_4_ nanocomposites with 50% SGCN demonstrate the most efficient degradation of RY-18 which is 83% at pH 3. Furthermore, the SGCN-based nanocomposites showed the highest antibacterial performance among all synthesized materials, highlighting their potential for multifunctional environmental applications.

## Data Availability

The original contributions presented in the study are included in the article/supplementary material, further inquiries can be directed to the corresponding authors.
